# How Big Is It Really? Assessing the Efficacy of Indirect Estimates of Body Size in Asian Elephants

**DOI:** 10.1371/journal.pone.0150533

**Published:** 2016-03-03

**Authors:** Simon N. Chapman, Hannah S. Mumby, Jennie A. H. Crawley, Khyne U. Mar, Win Htut, Aung Thura Soe, Htoo Htoo Aung, Virpi Lummaa

**Affiliations:** 1 Department of Animal and Plant Science, University of Sheffield, Western Bank, Sheffield, S10 2TN, United Kingdom; 2 Department of Zoology, University of Cambridge, Downing Street, Cambridge, CB2 3EJ, United Kingdom; 3 Myanma Timber Enterprise, Extraction Department, Ministry for Environmental Conservation and Forestry, Yangon, Myanmar; 4 Department of Biology, University of Turku, FIN-20014, Turku, Finland; University of Alabama at Birmingham, UNITED STATES

## Abstract

Information on an organism’s body size is pivotal in understanding its life history and fitness, as well as helping inform conservation measures. However, for many species, particularly large-bodied wild animals, taking accurate body size measurements can be a challenge. Various means to estimate body size have been employed, from more direct methods such as using photogrammetry to obtain height or length measurements, to indirect prediction of weight using other body morphometrics or even the size of dung boli. It is often unclear how accurate these measures are because they cannot be compared to objective measures. Here, we investigate how well existing estimation equations predict the actual body weight of Asian elephants *Elephas maximus*, using body measurements (height, chest girth, length, foot circumference and neck circumference) taken directly from a large population of semi-captive animals in Myanmar (*n* = 404). We then define new and better fitting formulas to predict body weight in Myanmar elephants from these readily available measures. We also investigate whether the important parameters height and chest girth can be estimated from photographs (*n* = 151). Our results show considerable variation in the ability of existing estimation equations to predict weight, and that the equations proposed in this paper predict weight better in almost all circumstances. We also find that measurements from standardised photographs reflect body height and chest girth after applying minor adjustments. Our results have implications for size estimation of large wild animals in the field, as well as for management in captive settings.

## Introduction

The size of an organism has a major role in its life-history, influencing aspects such as mating behaviour [1], reproduction [2,3], offspring mass [4] and dominance [5,6]. Knowledge of an individual’s size can also be used to tailor medical treatment or intervention, to aid conservation efforts [2,7,8], detect the extent of population declines [9,10], and for studies on the behaviour, ecology and evolution of animals [7,11,12]. For wild, large or dangerous animals, however, this data may be difficult to obtain [13–17], especially as immobilisation may negatively affect the animal [18].

In these cases, size can be measured indirectly. There is already a diverse array of methods through which linear size measurements can be estimated, including photogrammetric methods [5,8,15,16,19–21], use of acoustic signals [22–24], using footprint size [25] or brood mass [2], and very basically by observer estimations [9,13,26]. Weight is an especially important measure of body size; not only does it play a role in the traits already listed, but it also is a marker of body condition [27]. It is, however, often even less feasible to obtain a measure of an animal’s weight than its height or length, especially for larger species [17]. Whilst body parameters such as height, girth and length can quickly and easily be measured on immobilised animals if required [8,15,28], weighing large animals, particularly mammals, requires equipment that may not be practical to use in the field [15]. Measurements of these other, more easily obtained, body parameters are frequently used in equations to predict weight [5,14,28–30], such as using chest girth to estimate weight of polar bears *Ursus maritimus* [28]. Other techniques, though less commonly used, include estimation from faecal bolus weight [31,32] or the size of teeth [33].

Though these techniques are frequently used in lieu of direct measurements, their accuracy cannot always be properly assessed, often because of the difficulty in obtaining these measurements directly. For instance, in sperm whales *Physeter macrocephalus*, acoustically ascertained lengths are validated with photogrammetric lengths [22,23], but this has shortcomings, most notably the difficulty in linking acoustically obtained size to a specific individual when more than one whale is present [10]. Photogrammetric techniques rely on calibration to a scale of known size, and this is often used to validate the accuracy of the method in place of direct comparison between photographic and actual measurements [16,19–21]. A range of prediction equations based on measurements have been developed to estimate animal weight, though to what extent these prediction equations for body weight are suited to a specific population, rather than the species as a whole, is often unknown–their applicability to populations beyond the individuals included in the original studies has rarely been tested.

The Asian elephant *Elephas maximus* is an ideal species with which to test the accuracy of predictive tools for a number of reasons: size may be linked to traits such as male dominance [34], conservation is of vital importance in this species (the Asian elephant is classified as endangered), and elephants can be dangerous and immobilisation undesirable [35], so lack of body size data is often an issue. To counter the latter problem, numerous equations exist for the species that predict weight based on other body parameters [36–40], and photogrammetry has recently been used to obtain length measurements [41]. Though photogrammetry is often used to obtain measures of height in other elephant species [32,42,43], it has not been validated with directly obtained measures in Asian elephants and a study of semi-captive elephants provides an opportunity to do so for the first time. We also estimate both weight and height from photographs and test their accuracy at predicting real body size, as these parameters are important in distinguishing between growth and increasing weight alone [34].

In this study, we aim to assess the efficacy of existing prediction equations for estimating weight by using a large semi-captive population of known age Asian timber elephants in Myanmar, for which cross-sectional and semi-longitudinal weight and morphometric measurements (height, chest girth, neck girth, foot circumference and back length) are available, allowing us to directly compare predicted to actual values. In addition, we develop sex-specific equations to allow individually-tailored veterinary care for elephants of all ages, for example to enable the application of more accurate doses of medication when dosage is weight-dependent. We also test how body height and chest girth from photographs of individual elephants correlate to directly measured height and chest girth. In combination, our study forms an extensive and detailed appraisal of the use of direct and photogrammetric methods to accurately estimate the height and weight of a larger-bodied mammal.

## Methods

### Study Population

Asian elephants are an endangered species [44], present in discontinuous populations across South East Asia and the Indian sub-continent. The second largest wild population of Asian elephants is found in the Union of Myanmar, with as many as 5,000 individuals [45,46]. Myanmar also has the largest captive population, of around 5,000 elephants [46], 2,700 of which are government-owned through the Myanma Timber Enterprise and are used for sustainable logging [47]. Historically the captive-born population has been supplemented with elephants captured from the wild [48], to the extent where almost half of the living elephants were born in the wild. The logging elephants are released into the forest at night for up to fourteen hours to forage at will and mate unsupervised [48]; both wild and captive bulls have access to oestrus females. As there is no additional supplementation or selective breeding, they are classed as a semi-captive population.

Every elephant is marked with a unique identification (ID) number, and has important life-history information recorded in log books [49], including information on health, mother ID, body measurements taken by vets at each health check, and dates of birth and death. For wild-born individuals, age is not accurately known, so year of birth is visually estimated upon capture based on numerous body condition markers: facial concavity, skin pigmentation, ear folding, and size (though this only applies for younger individuals) [50]. This population is therefore ideal for testing prediction tools.

Infants stay at the mother’s heel until the age of 5, at which point they are separated and trained. The work season lasts from mid-June to mid-February, with a rest period during October. This working season is timed to coincide with the monsoon (July-October) and cool (November-February) seasons, so that no work is done during the dry season (March-June) [51], when temperature-related mortality is highest [52]. Elephants have well-defined workload limits each year, which cannot be exceeded. As well as an annual maximum tonnage of logs each elephant can move, there are strict limits to daily and weekly work–in 2010 these limits were set to a daily maximum of eight hours, with a break at noon, and no more than five days of work in a week [53]. Elephants are only used for light work from age 5 years until 17, becoming part of the true working population after this. The main working population are able to drag logs and engage in heavier work, continuing up until retirement at around 55 years old [54]. Pregnant females are given time off work from mid-pregnancy through to a year after birth [48], and are then given lighter work up until weaning, at which point the mother begins heavier work again.

### Data Selection

The weight of elephants and five morphometric measures—back length (from the base of the neck to the fold of the tail), chest girth (measured behind the forelegs with care taken to ensure it was not affected by inhalation by the elephant), foot circumference (right forefoot), height (from scapula to the ground) and neck circumference (taken close to the body, with care taken that the measurement was not affected by breathing or any ropes or bells worn by the animal)—were recorded in camps in five locations in Myanmar: Pyinmana, Monywa, East Katha, West Katha and Kawlin. Weight was measured to the nearest 1kg using EziWeigh 3000 scales, whilst the other measurements were taken with tape measures, and were recorded either in inches or centimetres, depending on location. A single trainer trained a measurer at each camp and observed them taking at least one measurement of each individual herself; repeated monthly measures were then taken by the measurer independently. For consistency, all elephants were measured in the morning on non-work days. Individuals in the final year of pregnancy were not included in the analyses. All measurements were converted into centimetres during collation of data for consistency, though this means measurements are only correct to the nearest multiple of 2.54cm (i.e. 1 inch). A total of 404 elephants aged between 9 months and 71 years were included in this study, with each individual having one or more measures of weight and at least one other body parameter measured. Of these, 224 were female and 180 were male.

In Pyinmana (*n* = 72), individuals were measured on a monthly basis between December 2011 and October 2012. Elephants were recorded cross-sectionally just once in Monywa in 2012 (*n* = 69). In East Katha (*n* = 71), data were collected monthly from December 2012 until the end of 2013. In West Katha (*n* = 45), monthly measurements have been taken since June 2012, with data up until March 2015 available. Kawlin (*n* = 147) data have been collected since November/December 2012, with records up until April 2015 available. However, for camps where monthly measurements had been taken, there are some months with missing data. Multiple measurements of the same individuals, which can be averaged, are likely to reduce variation in the measurements due to feeding patterns, illness and work schedules.

In addition to the body measurement data, photographs of 151 elephants in profile were taken in Monywa, East Katha, West Katha and Kawlin, with a measuring stick of known length held up directly against them. One photo was selected for each elephant, based on three criteria: whether the elephant was perpendicular to the camera, whether the ends of the scale were visible, and whether the foreleg was straight. Using ImageJ software version 1.48 [55], the 'photographic' heights of elephants were ascertained. This was done by using the visible measuring stick to set the scale for each photo, and then measuring the shoulder height from scapula to the base of the foot. If the leg was straight but not completely vertically orientated, the measurement would be taken from the scapula to the area of ground level to the base of the foot. A proxy for chest girth was also measured on the photographs: from the underside of an elephant behind the leg in a vertical line to the corresponding point on the elephant’s back. Measurements were independently taken by four people, to test if the method can consistently be applied. This research has ethics approval from the University of Sheffield, Department of Animal and Plant Sciences, Department Ethics Committee. Fieldwork permission was granted from the Ministry of Environmental Conservation and Forestry in Myanmar.

### Statistical Analyses

All analyses were conducted using R version 3.2.1 [56].

#### Predicting weight

To test the ability of previously published growth equations (Table 1) to accurately estimate the weight of elephants using different available body parameters, the equations were first applied to subsets of the data which included only those elephants that had weight and the other body parameters specified in the equation recorded for them. When there were many equations in a published study, only those reported to fit well were selected. Furthermore, only elephants within the original age range of each equation were used, with those outside the range excluded from testing for that particular equation. The predicted values of weight obtained from this procedure were averaged for each individual, and the observed monthly weights were averaged for each individual, giving one predicted and one actual value for each elephant. This allowed us to approximate the cross-sectional nature of the original studies as closely as possible. There were two exceptions to this: the two equations from Sukumar et al. [38], which were created from measurements taken over a period of four years. These predicted values were then run in linear regression equations against the actual weights of the elephants, with a separate regression for each equation.

**Table 1:** Existing equations for predicting elephant weight from the literature.

Equation	Age Range	Sex	Reference
-3336 + 18 x Chest Girth	1–57^1^	Both	[37]
-3408 + 17.9 x Chest Girth	1–13	Both	[37]
-2481 + 15.5 x Chest Girth	18–28	Both	[37]
-3786 + 19.4 x Chest Girth	29–39	Both	[37]
-4249 + 20.8 x Chest Girth	40–57	Both	[37]
-1776.5 + 1937.3 x Neck Circumference	0–57^2^	Both	[40]
-2493.4 + 1484 x Chest Girth	0–57^2^	Both	[40]
-2304 + 1427.6 x Chest Girth	0–10^2^	Male	[40]
-2498.9 + 1427.6 x Chest Girth	0–10^2^	Female	[40]
-2222.2 + 1427.6 x Chest Girth	10–20^2^	Male	[40]
-2417.1 + 1427.6 x Chest Girth	10–20^2^	Female	[40]
-2078.64 + 1427.6 x Chest Girth	20+^2^	Male	[40]
-2273.6 + 1427.6 x Chest Girth	20+^2^	Female	[40]
(Chest Girth^2^ x Height)/10000	0–6	Both	[36]
(0.98 x Chest Girth^2^ x Height)/10000	6+	Female	[36]
(0.93 x Chest Girth^2^ x Height)/10000	6+	Male	[36]
-5088 + 22.87 x Chest Girth	15–50	Both	[39]
-4920 + 25.49 x Length	15–50	Both	[39]
-1010 + 0.036(Length x Chest Girth)	15–50	Both	[39]
3255(1—exp(-0.149(Age + 3.16)))^3^	2–60	Male	[38]
3055(1—exp(-0.092(Age + 6.15)))^3^	2–70	Female	[38]

^1^ No elephants aged 13–18 were used for the original equation

^2^ Measurement in metres

To select the best fitting equations, we assessed values of *R*^*2*^ and the mean and standard deviation of predicted weights relative to actual weights, represented here as a percentage of actual weight, with a value of 100% showing predicted and actual measures to be the same. *R*^*2*^ by itself is insufficient to inform on the accuracy of these equations [57]–it is a measure of relation, rather than agreement–whereas relative mean is directly linked to how close predicted weights are to actual weights–a representation of agreement.

#### New equations for predicting weight

In order to create new equations that best predict weight from the available body parameters (height, chest girth, neck circumference, foot circumference and length) and in this specific population, we first explored the correlations separately between each of the different body parameters and weight for each sex. The individual body parameter correlates most closely related to weight were used in linear regression mixed-effects models using the function *lmer* from the package *lme4* version1.1–9 [58]. Separate models were created for each sex, as growth curves show large differences in the pattern of weight gain and values of weight between the sexes [34]. Different body parameters were not used in combination in any of the equations; as they are closely correlated with each other they should not be included in a single model—they add little in the way of accuracy, whilst adding a degree of complexity that would make them less applicable in the field [17].

Initially, a series of full models were created using a training set of 70% of the dataset (*n* = 188 females and 155 males). The data were split by observation rather than individual to ensure a range of ages were present in the training dataset and that a few young elephants with only a single row of data each would not affect the results. The fixed effects in these full mixed-effects models were as follows: the body parameter (height, chest girth, neck circumference, foot circumference and length), with the closest correlates being height and weight; age in years, to account for the difference between age-related increases in the body parameter and weight; age squared, to allow non-linear increases in weight; and season of measurement, which was a factor with three levels (Dry, Cool, Monsoon) to account for seasonal variation in body condition and weight [27]. There were two random effects: elephant ID, a factor with *n* levels, with *n* being the number of elephants in that particular subset, which was included to account for between individual variation, and location, a factor with five levels (West Katha, East Katha, Pyinmana, Monywa, Kawlin), to account for variation by region. As the variance in the age of wild-born elephants is likely to be greater than a year, they were not used in the construction of the models in which age was present (in these models, *n* = 131 captive-born males and 132 captive-born females—subset from the training data). We also constructed linear models without random effects, but with the same fixed factors as above—to be used as prediction equations in the field—and tested their fit to the data in comparison to the models with random terms to determine how much individual-level variation and location-level variation alters the model. We then produced reduced linear models, which excluded both age and the random effects, to allow application to all elephants, regardless of birth origin. Finally, prediction equations were extracted from the models and tested on a testing subset of the data, which comprised the remaining 30% of the dataset (*n* = 139 females and 121 males). This training and testing procedure was appropriate for this dataset, with repeated measures for individuals being treated as independent and, in the mixed-effects models, the random ID term statistically controlling for between-individual variation.

Prediction equations that fitted well with the testing subset, based on regression outputs (S1 Table), were then used to obtain predictions of weight from the entire dataset. These predicted weights were compared to the actual measured weights of elephants through calculating the mean and standard deviation of predicted weights relative to actual weights and from the outputs of linear regressions—regressing observed weight against predicted weight—to test for applicability in the field. The fits of all these equations were then compared with the fits of the previously published equations (which were also compared to the whole dataset), to allow direct comparison. No values were averaged for this analysis. If an existing equation was applicable to both sexes, it was run separately on both.

#### Predicting height and chest girth

In order to test the photographic method of estimating height and chest girth, estimates of these parameters obtained from analysis of photographs were compared to the actual measurements of the animals taken in the field. To test for differences in values obtained through the ImageJ method by different people, Pearson’s product moment correlation coefficients were calculated. Linear regressions were then run between actual measurements and the heights and proxy measurements for girth obtained using ImageJ software.

## Results

**Fig 1:**
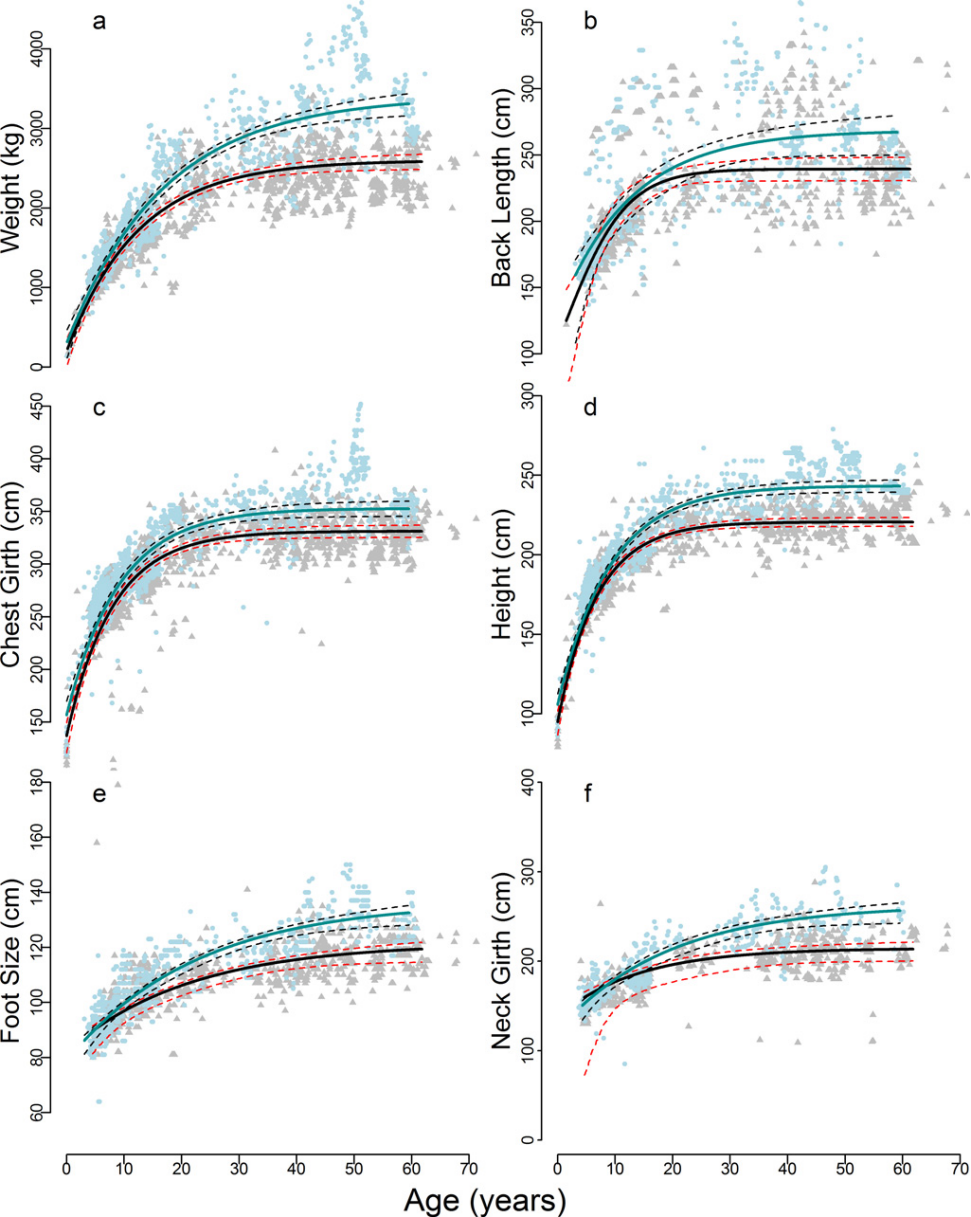
Size of elephant body parameters during life (0–70 years). a) weight in kilograms; b) back length; c) height; d) chest girth; e) foot circumference; f) neck circumference. Blue points and lines represent males, grey points and black lines represent females. 95% confidence intervals are shown as dashed black lines for males and dashed red lines for females, and were calculated from Monte Carlo simulations. Excluding weight, all measurements are in centimetres.

Measurements in elephants, in fitting with standard mammalian growth patterns, differ with age and sex (Fig 1), and appear in this population to represent determinate growth [34]. Males are larger than females of the same age in every body parameter within a few years of birth, and continue to gain weight throughout life.

### Existing Prediction Equations

**Fig 2:**
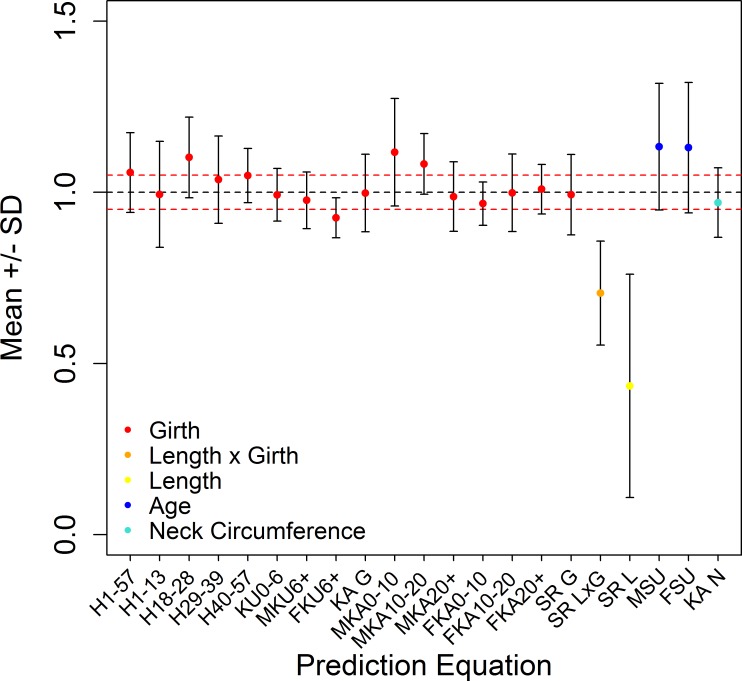
Mean and standard deviations of predicted weights from equations relative to actual weights. Grey bars represent the entire range of the equation and the red dashed line represents 5% above and below one. Colour of circles indicate which parameter was used in the equation. Letter codes in the x-axis labels are as follows: H = Hile et al. [37]; KU = Kurt and Garai [36]; KA = Kanchanapangka et al. [40]; SR = Sreekumar and Nirmalan [39]; SU = Sukumar et al. [38]; G = Chest Girth; L = Length; N = Neck Circumference; F = Female only; M = Male only. Numbers represent the age range.

We applied twenty-one prediction equations from five previous studies in the literature to data on the timber elephants in order to test whether they are applicable in the field for a different population than the one from which they were created. *R*^*2*^ from these models ranged from 0.080 to 0.956 for these prediction equations, highest from Kurt and Garai’s [36] male equation (*R*^*2*^ = 0.956), which uses chest girth and height, and lowest from Sreekumar and Nirmalan’s [39] length-based equation (*R*^*2*^ = 0.080). The Kurt and Garai [36] equation for infants provides the most accurate fit (*R*^*2*^ = 0.814, mean±SD (%) = 99.3±7.7), though this only applies for elephants under the age of 6 (Fig 2, Table 2).

**Table 2:** Summary of the fit of equations from the literature to the Myanmar timber elephant dataset.

Equation	*N*	*R*^*2*^	Mean±SD (%)
Hile et al. (1–57)	380	0.869	105.8 ± 11.6
Hile et al. (1–13)	114	0.718	99.4 ± 15.5
Hile et al. (18–28)	72	0.651	110.2 ± 11.8
Hile et al. (29–39)	53	0.286	103.7 ± 12.8
Hile et al. (40–57)	104	0.806	104.9 ± 7.9
Sreekumar and Nirmalan (Girth)	232	0.680	99.3 ± 11.8
Sreekumar and Nirmalan (Length x Girth)	190	0.349	70.5 ± 15.2
Sreekumar and Nirmalan (Length)	193	0.08	43.4 ± 32.6
Sukumar et al. (Female)	218	0.722	113.0 ± 19.1
Sukumar et al. (Male)	183	0.788	113.3 ± 18.5
Kanchanapangka et al. (Girth)	380	0.864	99.8 ± 11.3
Kanchanapangka et al. (Neck Circumference)	141	0.880	97.0 ± 10.1
Kanchanapangka et al. (Girth; Male; 0–10)	47	0.488	111.7 ± 15.7
Kanchanapangka et al. (Girth; Female; 0–10)	37	0.921	96.7 ± 6.3
Kanchanapangka et al. (Girth; Male; 10–20)	43	0.840	108.3 ± 8.9
Kanchanapangka et al. (Girth; Female; 10–20)	43	0.488	99.8 ± 11.3
Kanchanapangka et al. (Girth; Male; 20+)	92	0.571	98.7 ± 10.2
Kanchanapangka et al. (Girth; Female; 20+)	154	0.668	100.9 ± 7.2
Kurt and Garai(0–6)	80	0.814	99.3 ± 7.7
Kurt and Garai(Female; 6+)	221	0.873	97.7 ± 8.3
Kurt and Garai(Male; 6+)	164	0.956	92.6 ± 5.8

For the actual equations, see Table 1.

### New Prediction Equations

We found that weight was highly correlated in both sexes with chest girth (male *r* = 0.949, *n* = 177, *p* <0.001; female *r* = 0.916, *n* = 221, *p* <0.001) and height (male *r* = 0.954, *n* = 178, *p* <0.001; female *r* = 0.913, *n* = 223, *p* <0.001); these parameters were selected for use in the models. Neck circumference was also highly correlated, but less so in females than males (male *r* = 0.938, *n* = 65, *p* <0.001; female *r* = 0.837, *n* = 82, *p* <0.001), and fewer elephants had available data on neck circumference. In contrast, foot circumference (male *r* = 0.888, *n* = 162, *p* <0.001; female *r* = 0.641, *n* = 189, *p* <0.001) and length (male *r* = 0.702, *n* = 162, *p* <0.001; female *r* = 0.678, *n* = 197, *p* <0.001) predicted weight considerably more poorly, especially for females. Model comparison showed chest girth to be the best single predictor of weight in both sexes (Table 3; see S2 and S3 Tables for model parameters). The ΔAIC values indicate that the model including height and chest girth is better-supported than either chest girth or height alone. However, as discussed in the methods, any additional accuracy is counteracted by an increase in complexity that would make field use less practical, and the correlation between the body parameters makes it difficult to interpret models including more than one parameter. Therefore, chest girth is the preferable single measurement for use in the equations. The most parsimonious model has the terms chest girth, age and age^2^. Season of measurement had no significant effect on predicted weight in either males (*χ*^*2*^_2_ = 1.13, *p* = 0.569) or females (*χ*^*2*^_*2*_ = 2.06, *p* = 0.357) in this sample.

**Table 3:** Comparison of linear mixed-effects models predicting weight from other measurable body parameters.

Model	Sex	AIC	ΔAIC	Log-likelihood	*χ*^*2*^	d.f.	*p*
**Chest Girth only**	**Male**	**8323.1**	**30.1**	**-4154.6**	**32.06**	**1**	**<0.001**
Height only	Male	8428.7	135.7	-4207.4	137.68	1	<0.001
**Chest Girth only**	**Female**	**8973.7**	**23.6**	**-4479.9**	**25.60**	**1**	**<0.001**
Height only	Female	8981.6	31.5	-4483.8	33.45	1	<0.001

The best-supported model has the lowest AIC value and is shown in bold. ΔAIC values are the difference between the model in the table and the model *~Chest Girth + Height + Age + Age*^*2*^. Analysis was performed on 650 observations of 127 males, and on 695 observations of 130 females.

The comparison of training and testing results is provided in S1 Table. All prediction equations below are based upon models created with the training dataset.

The predictions of weight obtained from the mixed-effects models are accurate, predicting values very close to the actual weights of the individuals in the model (male *R*^*2*^ = 0.986, mean±SD (%) = 100.3±5.8; female *R*^*2*^ = 0.963, mean±SD (%) = 100.3±5.5). As random effects cannot be included in an equation, field-applicable equations were derived from the basic linear regression models (S4 Table). The equations for chest girth are as follows, where *W* = weight, *CG* = chest girth and *t* = age:

$$\text{Male}\;W=-1828+11\times CG+35\times t-0.22\times t^{2}$$ (1)

Male(reduced)W=−3636+18.7×CG (2)

$$\text{Female}\;W=-1539+9.9\times CG+36.5\times t-0.43\times t^{2}$$ (3)

Female(reduced)W=−2562+15.0×CG (4)

**Fig 3:**
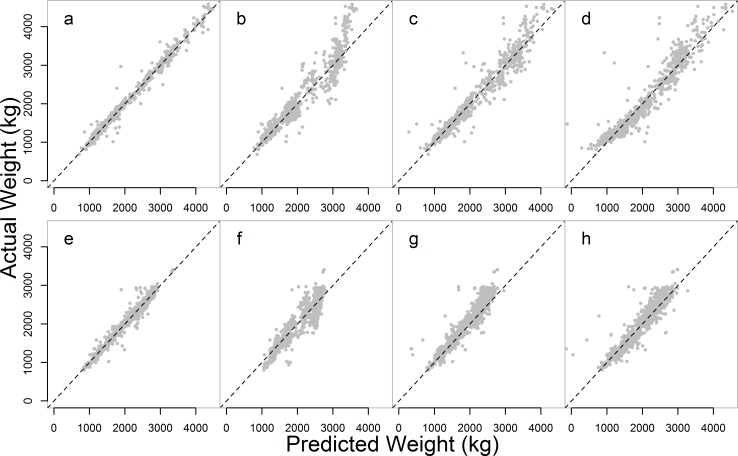
Comparison of fit of predicted values from different equations. a) male mixed-effects model; b) equation derived from mixed-effects model for males; c) equation derived from linear regression model for males (Eq 1); d) equation derived from linear regression model without age terms for males (Eq 2); e) female mixed-effects model; f) equation derived from mixed-effects model for females; g) equation derived from linear regression model for females (Eq 3); h) equation derived from linear regression model without age terms for females (Eq 4). The black line is 1:1 predicted weights to actual weights. Points above the line are underestimated, and those below are overestimated.

When random terms accounting for ID and camp were removed, the fit was slightly poorer than for the mixed-effects models, as expected (male *R*^*2*^ = 0.944, mean±SD (%) = 101±10.2; female *R*^*2*^ = 0.873, mean±SD (%) = 101.6±10.9). However, there was little difference between this test of known-age captive-born elephants only and that for all elephants, including age-estimated wild-born individuals (male *R*^*2*^ = 0.935, mean±SD (%) = 101±10.6; female *R*^*2*^ = 0.884, mean±SD (%) = 101.4±10.2). The reduced, chest girth only model with no age term, to be used in populations of elephants of unknown ages, has a larger deviation (male *R*^*2*^ = 0.903, mean±SD (%) = 100.3±14.8; female *R*^*2*^ = 0.839, mean±SD (%) = 101.5±13.2), though it is better at prediction at higher weights than the equations derived from the mixed-effects model (Fig 3).

**Fig 4:**
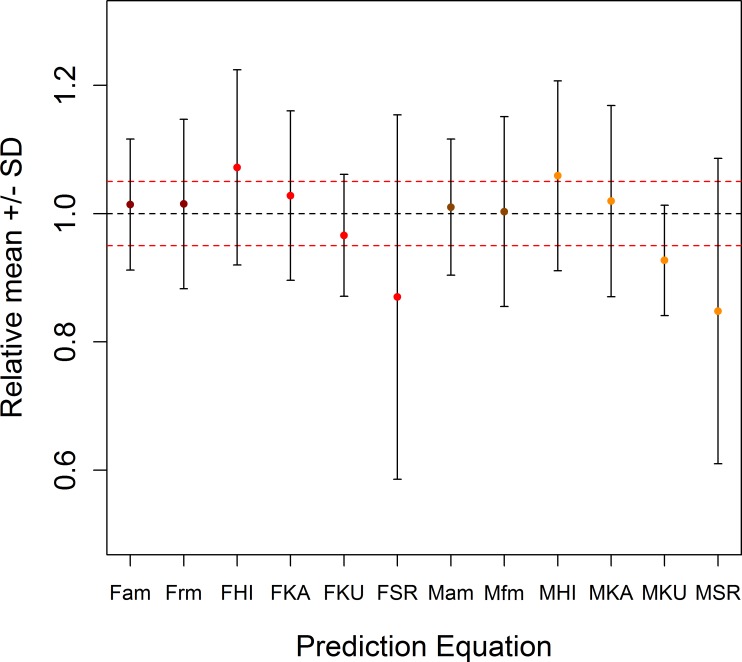
Mean and standard deviations of new and previously published prediction equations. Grey bars represent the entire range of the equation and the red dashed line represents 5% above and below one. Letter codes in the x-axis labels are as follows: F = Female; M = Male; KU = Kurt and Garai [36]; HI = Hile et al. [37]; KA = Kanchanapangka et al. [40]; SR = Sreekumar and Nirmalan [39]; am = Age model equations (Eqs 1 and 3); rm = Reduced model equations (Eqs 2 and 4). Red circles represent females, and orange circles males. Darker circles indicate the equations proposed in this paper.

The new equations for the Myanmar elephants with age terms were more effective for predicting weight than any of the previously published equations (Fig 4, S5 Table). The reduced equations with chest girth as the sole predictor were also preferable for predicting weight. However, for young elephants, the Kurt and Garai [36] <6 years old equation (female *R*^*2*^ = 0.815, mean±SD (%) = 97.8±6.0; male *R*^*2*^ = 0.616, mean±SD (%) = 99.1±9.5) was of a similar efficacy to the female equation (Eq 3) applied to only those under 6 years (*R*^*2*^ = 0.745, mean±SD (%) = 102.9±7.5), and more accurate than the male equation (Eq 1) applied to the same age group (*R*^*2*^ = 0.553, mean±SD (%) = 99.4±10.2). The reduced equations were also less accurate when applied to young elephants (female *R*^*2*^ = 0.691, mean±SD (%) = 106±9.6; male *R*^*2*^ = 0.542, mean±SD (%) = 96.1±18.2).

### Predicting Height and Chest Girth

**Fig 5:**
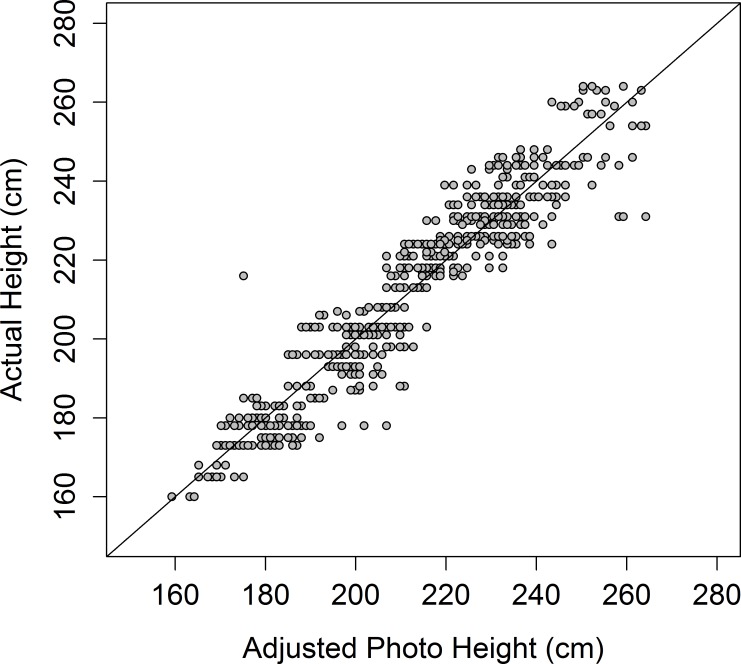
Predicted heights from photos (*n* = 151). Photographic heights were adjusted by the equation 10.8 + 0.99 x *Photo Height*. Dashed line represents 1:1 estimate:actual height.

**Fig 6:**
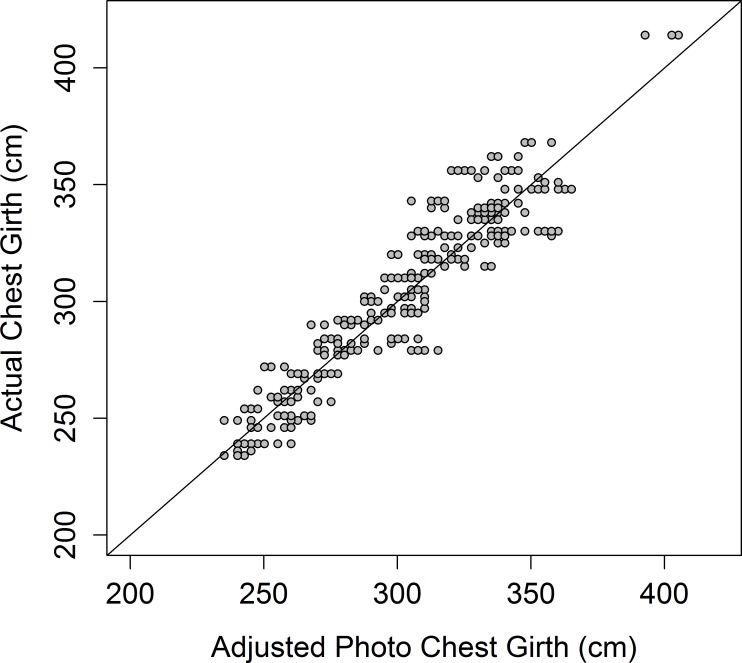
Predicted chest girth from photos (n = 78). Photographic measurements were adjusted by the equation 25.2 + 2.5 x *Photo Girth*. Dashed line represents 1:1 estimate:actual chest girth.

**Fig 7:**
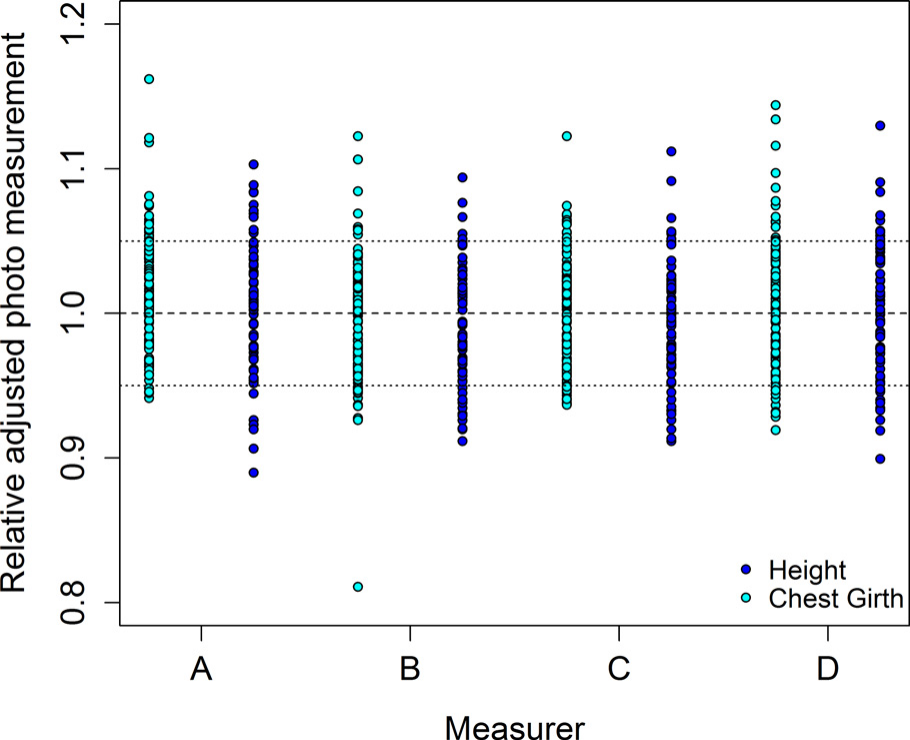
Adjusted photographic measurements relative to actual measurements of elephants, separated by measurer. Photographic heights (blue points) were adjusted by the equation 10.8 + 0.99 x *Photo Height*; photographic chest girths (cyan points) were adjusted by the equation 25.2 + 2.5 x *Photo Girth*. The long-dashed line represents actual heights. Short-dashed lines represent 5% above and below actual heights.

The accuracy of using photographs to estimate an elephant’s height and chest girth is high (Figs 5 and 6), with the values predicted from the photographs being highly correlated with the measured heights (*r* = 0.946) and chest girths (*r* = 0.940). There was little difference in values obtained by different people measuring height and chest girth from photographs: *r* was greater than 0.97 in all cases (S6 Table). Most height values are underestimated by a similar amount—between ~5 and 20cm—meaning a simple equation can be used to obtain heights closer to the actual value (Fig 7):

10.8+0.99×PhotoHeight (5)

Similarly, the proxy photo measurements of chest girth can be adjusted for estimated values close to the actual chest girth of elephants (Fig 7):

25.2+2.5×PhotoGirth (6)

## Discussion

Weight is a particularly important measure of size, serving as an indicator of body condition [27] as well as influencing other traits [3–5], whilst height and/or length is more indicative of skeletal size [34]. It is, however, more difficult to directly measure weight than other body parameters, so prediction equations are frequently used, though often without validation using known values of weight. We found chest girth, sometimes referred to as heart girth or thorax circumference in the literature, to be the best predictor of weight in Asian elephants, in line with previous studies on large mammals [14,17,28,59–61], and some of the other weight prediction equations for Asian elephants [36,37,39]. Whether the relationship between chest girth and body weight, and thus the equation used for predicting weight from chest girth, differs between distinct populations of a species is not known outside a few species, and there is no consensus: white-tailed deer *Odocoileus virginianus* populations differ in this relationship [59], though populations of the grizzly bear *Ursus arctos* may not [62]. Similar to Weckerly et al. [59], we found that equations from other populations of Asian elephants did not fit the Myanmar timber elephant dataset particularly accurately; only the Kurt and Garai [36] equation for elephants specifically under age 6 was more accurate than the new equations we propose in this paper, possibly due to lower variation in body weight in early life [38]. Future work on animal species in which weight is predicted from equations should therefore exercise caution, rather than using established, but untested, equations for predicting weight. We have also shown that although mixed-effects models provide the best predictions of weight by taking individual variation into account, their actual application in field studies are limited: random effects (in this case ID and location) cannot, by their very nature, be included in a single, simple equation.

For indirectly measuring linear body measurements, such as height or length, photogrammetry is used, especially for large animals [8,10,41]. As with weight, measures obtained in this manner have rarely been tested against actual values. By comparing actual heights with heights estimated through photogrammetry, we have shown that a basic photogrammetric method is sufficiently accurate for estimation of height, and is likely to be useful for estimation of other linear morphometric measurements. We have also shown that a proxy measurement for chest girth is highly correlated with actual chest girth and that these photogrammetric measurements could therefore be used for prediction of weight. This may allow estimation of the weight of wild elephants without sedation. The difference in measurement values obtained by different measurers was negligible; basic photogrammetry of this kind can therefore be easily implemented, with little training required. We found that the accuracy of this method is high, without the need for high-end equipment such as lasers, which have been used in recent photogrammetric studies [19,20]. The main challenge of this basic technique, however, is trying to include a scale directly next to an animal with which measurements can be calibrated–for this population of semi-captive elephants the scale is held directly against an individual, but for wild or dangerous species other solutions will have to be found. Furthermore, though a scale is required for calibration, it is not sufficient for validation, as the technique does not guarantee accuracy; a simple equation, calculated from validation with actual values, may be required for increasing the accuracy of further measurements collected with photogrammetry [32].

For the Myanmar timber elephants, the prediction equation most commonly used in the field by local veterinarians is the Hile et al. [37] equation for all elephants, though this tends towards overestimation (*R*^*2*^ = 0.869, mean±SD (%) = 105.8±11.6), and as such is ill-suited for accurate prediction of weight in this population. Of the equations proposed in this paper, the new equations we have produced including age provide very good fits, even with wild-born elephants included. The inclusion of age terms does, however, limit their applicability as management tools to elephants of known age, as wild-born elephants cannot be guaranteed to have ages estimated to within a year of their actual age. For wild-born elephants captured later in life where age cannot be accurately estimated, the new reduced equations (Eqs 2 and 4) we have produced should be used instead of previous equations. This could be because wild-caught elephants have growth patterns that are different to captive-born elephants [38], even if they are aged accurately. If the equations with age are too complex for field application, the reduced equations are suited for all elephants over age 6. Even though these new equations are more accurate over the entire population, the Kurt and Garai [36] young elephant equation (Table 1) provided the best accuracy for calves aged under 6.

For the purposes of conservation, knowing body size is critical. Size is of great ecological importance: at a species level, body size can dictate niche differentiation [63], population density [64] and range size [65]; at an individual level, body size can influence resource choice and trophic level [66], metabolic rate [67] and intraspecific variation in range size [11]. Accurate measurements of body weight are vital for practical management: dosages of both medicinal and immobilising drugs often depend on weight [35,40,68–71], which can be used for veterinary care, collection of other body data, and for fitting GPS collars [68]. Ideally animals will be weighed on scales, though this is not always possible, either because of logistical issues or the difficulty of encouraging wild animals to stand on them; prediction equations, therefore, are often the only viable alternative. It is especially important to know the weight of Asian elephants as they are an endangered species, and this particular population is already unsustainable [47].

In conclusion, we have demonstrated that weight prediction equations provide a high enough level of accuracy that they can be used in the absence of scales, though they may be too specific to a certain population for use on other, distinct, populations of the same species without validation first. Basic photogrammetry can easily be implemented without specialist training and can also be highly precise, so long as a scale of known size is present and wholly visible, and that at least a small number of individuals are of known size, so that an appropriate equation can be used to obtain highly accurate measures of both weight and height.

## Supporting Information

S1 Table Fit of predictions on training and testing dataset. From linear models regressing observed weight against predicted weight.
(DOCX)

S2 Table Mixed-effects model estimates. Female *n* = 132 (720 observations); male *n* = 131 (689 observations). Estimates are for full models with all parameters included. The season estimates are in comparison to the cool season.
(DOCX)

S3 Table Model estimates for predicting body weight. Models included the following as explanatory variables: i) chest girth, age and season of measurement (female *n* = 132; male *n* = 131); ii) chest girth and age (female *n* = 132; male *n* = 131); iii) chest girth only (female *n* = 188; male *n* = 155). Intercepts are for the ‘Cool’ season, and are in kg. All models use a Gaussian distribution.
(DOCX)

S4 Table Regression equations for estimating weight from other body parameters. Where *P* is the body parameter.
(DOCX)

S5 Table Summary statistics of prediction equations. *R*^*2*^, intercept and slope were obtained from running linear models of actual weight against predicted weight. Relative mean and standard deviation are the mean and standard deviation of predicted weights as a percentage of actual weights.
(DOCX)

S6 Table Correlation coefficients between photo-measured heights and girths from different measurers. (DOCX)
