# Supplementary material for: How Big Is It Really? Assessing the Efficacy of Indirect Estimates of Body Size in Asian Elephants
Source: PLoS One. 2016 Mar 3;11(3):e0150533. doi: 10.1371/journal.pone.0150533 (PMC4777392; doi:10.1371/journal.pone.0150533)
Supplement: S1 Table — From linear models regressing observed weight against predicted weight. (DOCX) [file pone.0150533.s001.docx]

**Table S1. Fit of predictions on training and testing dataset.** From linear models regressing observed weight against predicted weight.

| Sex | Dataset | *n* | Equation | *R^2^* | Intercept | Slope |
| --- | --- | --- | --- | --- | --- | --- |
| Female | Training | 132 | -1539+9.9*CG+36.5*t*-0.43*t*^2^ | 0.877 | -13.84 | 1.060 |
| Female | Testing | 98 | -1539+9.9*CG+36.5*t*-0.43*t*^2^ | 0.857 | 83.12 | 0.942 |
| Female | Training | 188 | -2562+15*CG | 0.853 | 13.93 | 1.005 |
| Female | Testing | 139 | -2562+15*CG | 0.805 | 128.56 | 0.927 |
| Male | Training | 131 | -1828+11*CG+35*t*-0.22*t*^2^ | 0.945 | -4.87 | 0.997 |
| Male | Testing | 103 | -1828+11*CG+35*t*-0.22*t*^2^ | 0.942 | -14.50 | 1.013 |
| Male | Training | 155 | -3636+18.7*CG | 0.902 | 6.06 | 1.002 |
| Male | Testing | 121 | -3636+18.7*CG | 0.905 | 45.52 | 0.987 |
