# Supplementary material for: How Big Is It Really? Assessing the Efficacy of Indirect Estimates of Body Size in Asian Elephants
Source: PLoS One. 2016 Mar 3;11(3):e0150533. doi: 10.1371/journal.pone.0150533 (PMC4777392; doi:10.1371/journal.pone.0150533)
Supplement: S2 Table — Female n = 132 (720 observations); male n = 131 (689 observations). Estimates are for full models with all parameters included. The season estimates are in comparison to the cool season. (DOCX) [file pone.0150533.s002.docx]

**Table S2. Mixed-effects model estimates.** Female *n* = 132 (720 observations); male *n* = 131 (689 observations). Estimates are for full models with all parameters included. The season estimates are in comparison to the cool season.

|  | Male | | | Female | | |
| --- | --- | --- | --- | --- | --- | --- |
| Fixed Factor | Estimate | Std. Error | *t* value | Estimate | Std. Error | *t* value |
| Intercept | -580.57 | 134.33 | -4.322 | -62.27 | 129.59 | -0.480 |
| Chest Girth | 5.39 | 0.52 | 10.447 | 3.00 | 0.45 | 6.713 |
| Age | 77.18 | 6.52 | 11.837 | 80.92 | 6.16 | 13.146 |
| Age^2^ | -0.77 | 0.11 | -7.035 | -0.96 | 0.10 | -10.013 |
| Dry Season | -0.06 | 12.41 | -0.004 | 5.22 | 12.00 | 0.435 |
| Monsoon Season | -2.43 | 13.04 | -0.186 | -7.62 | 12.33 | -0.618 |
| Random Factor | Variance | Std. Dev |  | Variance | Std. Dev |  |
| ID | 47254 | 217.4 |  | 52729 | 229.63 |  |
| Camp | 6401 | 80.0 |  | 6118 | 78.22 |  |
| Residuals | 13671 | 116.9 |  | 13981 | 118.24 |  |
