# Supplementary material for: How Big Is It Really? Assessing the Efficacy of Indirect Estimates of Body Size in Asian Elephants
Source: PLoS One. 2016 Mar 3;11(3):e0150533. doi: 10.1371/journal.pone.0150533 (PMC4777392; doi:10.1371/journal.pone.0150533)
Supplement: S3 Table — Models included the following as explanatory variables: i) chest girth, age and season of measurement (female n = 132; male n = 131); ii) chest girth and age (female n = 132; male n = 131); iii) chest girth only (female n = 188; male n = 155). Intercepts are for the ‘Cool’ season, and are in kg. All models use a Gaussian distribution. (DOCX) [file pone.0150533.s003.docx]

**Table S3. Model estimates for predicting body weight.** Models included the following as explanatory variables: i) chest girth, age and season of measurement (female *n* = 132; male *n* = 131); ii) chest girth and age (female *n* = 132; male *n* = 131); iii) chest girth only (female *n* = 188; male *n* = 155). Intercepts are for the ‘Cool’ season, and are in kg. All models use a Gaussian distribution.

| Model | Sex | Parameter | Estimate | Standard Error | *t* | *p* |
| --- | --- | --- | --- | --- | --- | --- |
| i | Female | Intercept | -1535 | 86.68 | -17.704 | <0.001 |
|  |  | Chest Girth | 9.875 | 0.365 | 27.056 | <0.001 |
|  |  | Age | 36.48 | 2.724 | 13.392 | <0.001 |
|  |  | Age^2^ | -0.429 | 0.038 | -11.023 | <0.001 |
|  |  | Dry Season | -6.98 | 17.31 | -0.403 | 0.687 |
|  |  | Monsoon Season | -20.25 | 18.23 | -1.111 | 0.267 |
| ii | Female | Intercept | -1539 | 85.1 | -18.09 | <0.001 |
|  |  | Chest Girth | 9.857 | -0.363 | 27.18 | <0.001 |
|  |  | Age | 36.51 | 2.713 | 13.46 | <0.001 |
|  |  | Age^2^ | -0.429 | 0.039 | -11.06 | <0.001 |
| iii | Female | Intercept | -2562.105 | 62.216 | -41.18 | <0.001 |
|  |  | Chest Girth | 14.981 | 0.204 | 73.58 | <0.001 |
| i | Male | Intercept | -1831 | 96.55 | -18.963 | <0.001 |
|  |  | Chest Girth | 11.05 | 0.415 | 26.609 | <0.001 |
|  |  | Age | 34.39 | 3.335 | 10.312 | <0.001 |
|  |  | Age^2^ | -0.213 | 0.049 | -4.957 | <0.001 |
|  |  | Dry Season | -17.66 | 18.46 | -0.957 | 0.339 |
|  |  | Monsoon Season | -25.85 | 19.44 | -1.330 | 0.184 |
| ii | Male | Intercept | -1828 | 95.37 | -19.163 | <0.001 |
|  |  | Chest Girth | 10.97 | 0.408 | 26.861 | <0.001 |
|  |  | Age | 34.96 | 3.304 | 10.583 | <0.001 |
|  |  | Age^2^ | -0.220 | 0.048 | -4.546 | <0.001 |
| iii | Male | Intercept | -3636 | 69.380 | -52.41 | <0.001 |
|  |  | Chest Girth | 18.734 | 0.224 | 83.80 | <0.001 |
