# Supplementary material for: How Big Is It Really? Assessing the Efficacy of Indirect Estimates of Body Size in Asian Elephants
Source: PLoS One. 2016 Mar 3;11(3):e0150533. doi: 10.1371/journal.pone.0150533 (PMC4777392; doi:10.1371/journal.pone.0150533)
Supplement: S4 Table — Where P is the body parameter. (DOCX) [file pone.0150533.s004.docx]

**Table S4. Regression equations for estimating weight from other body parameters.** Where *P* is the body parameter.

| Body Parameter (*P*) | Regression Equation | |
| --- | --- | --- |
|  | Male | Female |
| Back Length | -722 + 12.7 x *P* | 50 + 8.7 x *P* |
| Chest Girth | -3636 + 18.7 x *P* | -2562 + 14.9 x *P* |
| Height | -3833 + 28.4 x *P* | -3315 + 25.7 x *P* |
| Foot Circumference | -3477 + 51.9 x *P* | -391 + 22 x *P* |
| Neck Circumference | -1992 + 20.5 x *P* | -1300 + 17.3 x *P* |
