# Supplementary material for: How Big Is It Really? Assessing the Efficacy of Indirect Estimates of Body Size in Asian Elephants
Source: PLoS One. 2016 Mar 3;11(3):e0150533. doi: 10.1371/journal.pone.0150533 (PMC4777392; doi:10.1371/journal.pone.0150533)
Supplement: S5 Table — R2, intercept and slope were obtained from running linear models of actual weight against predicted weight. Relative mean and standard deviation are the mean and standard deviation of predicted weights as a percentage of actual weights. (DOCX) [file pone.0150533.s005.docx]

**Table S5. Summary statistics of prediction equations.** *R^2^*, intercept and slope were obtained from running linear models of actual weight against predicted weight. Relative mean and standard deviation are the mean and standard deviation of predicted weights as a percentage of actual weights.

| Age Range | Sex | Equation | *R^2^* | Intercept | Slope | Mean ± SD (%) |
| --- | --- | --- | --- | --- | --- | --- |
| 0-71 | Female | Reduced | 0.839 | 38.03 | 0.977 | 101.534 ± 13.161 |
|  |  | Age | 0.884 | -1.35 | 0.995 | 101.436 ± 10.219 |
|  |  | Age (captive only) | 0.873 | 17.31 | 0.983 | 101.622 ± 10.889 |
|  |  | Kurt | 0.896 | 198.2 | 0.930 | 96.617 ± 9.506 |
|  |  | Hile | 0.838 | 251 | 0.814 | 107.240 ± 15.187 |
|  |  | Kanchan | 0.839 | -2.73 | 0.987 | 102.754 ± 13.246 |
|  | Male | Reduced | 0.903 | 18.60 | 0.997 | 100.293 ± 14.846 |
|  |  | Age | 0.935 | -21.23 | 1.010 | 100.974 ± 10.629 |
|  |  | Age (captive only) | 0.944 | -6.43 | 1.001 | 100.951 ± 10.234 |
|  |  | Kurt | 0.950 | 99.55 | 1.028 | 92.719 ± 8.611 |
|  |  | Hile | 0.903 | -151.17 | 1.036 | 105.889 ± 14.809 |
|  |  | Kanchan | 0.903 | -474.02 | 1.256 | 101.959 ± 14.934 |
| 0-6 | Female | Reduced | 0.691 | 203.81 | 0.751 | 105.973 ± 9.620 |
|  |  | Age | 0.745 | -43.89 | 1.020 | 102.935 ± 7.461 |
|  |  | Kurt <6 | 0.815 | 16.30 | 1.009 | 97.758 ± 6.046 |
|  | Male | Reduced | 0.542 | 721.21 | 0.378 | 96.057 ± 18.203 |
|  |  | Age | 0.553 | 440.52 | 0.618 | 99.405 ± 10.241 |
|  |  | Kurt <6 | 0.616 | 423.88 | 0.633 | 99.133 ± 9.546 |
| 6-71 | Female | Reduced | 0.821 | 56.59 | 0.969 | 101.390 ± 13.238 |
|  |  | Age | 0.871 | 3.79 | 0.993 | 101.387 ± 10.294 |
|  |  | Age (captive only) | 0.859 | 25.51 | 0.979 | 101.565 ± 11.013 |
|  |  | Kurt | 0.885 | 219.80 | 0.921 | 96.643 ± 9.600 |
|  |  | Hile | 0.821 | 267.79 | 0.807 | 107.471 ± 15.230 |
|  |  | Kanchan | 0.821 | 16.18 | 0.979 | 102.548 ± 13.301 |
|  | Male | Reduced | 0.885 | -39.78 | 1.020 | 101.163 ± 13.910 |
|  |  | Age | 0.920 | -48.94 | 1.020 | 101.296 ± 10.685 |
|  |  | Age (captive only) | 0.932 | -33.51 | 1.012 | 101.323 ± 10.204 |
|  |  | Kurt | 0.939 | 128.1 | 1.018 | 92.925 ± 8.558 |
|  |  | Hile | 0.885 | -213.61 | 1.061 | 105.639 ± 14.359 |
|  |  | Kanchan | 0.885 | -544.16 | 1.286 | 100.047 ± 14.538 |
