# Supplementary material for: How Big Is It Really? Assessing the Efficacy of Indirect Estimates of Body Size in Asian Elephants
Source: PLoS One. 2016 Mar 3;11(3):e0150533. doi: 10.1371/journal.pone.0150533 (PMC4777392; doi:10.1371/journal.pone.0150533)
Supplement: S6 Table — (DOCX) [file pone.0150533.s006.docx]

**Table S6. Correlation coefficients between photo-measured heights and girths from different measurers.**

| Measurer | *r* between measurers (Height) | | | *r* between measurers (Chest Girth) | | |
| --- | --- | --- | --- | --- | --- | --- |
|  | B | C | D | B | C | D |
| A | 0.975 | 0.984 | 0.981 | 0.984 | 0.980 | 0.975 |
| B |  | 0.978 | 0.973 |  | 0.989 | 0.980 |
| C |  |  | 0.984 |  |  | 0.980 |
